# Zika virus does not alter locomotor activity of Aedes albopictus (Diptera: Culicidae)

**DOI:** 10.11606/s1518-8787.2025059006968

**Published:** 2025-10-24

**Authors:** Pâmela dos Santos Andrade, Vivian Petersen, Antônio Ralph Medeiros-Sousa, Anderson Vicente de Paula, Paulo Roberto Urbinatti, Rosa Maria Marques de Sá Almeida, Tamara Nunes Lima-Camara

**Affiliations:** I Universidade de São Paulo Faculdade de Saúde Pública Programa de Pós-Graduação em Saúde Pública São Paulo SP Brazil Universidade de São Paulo. Faculdade de Saúde Pública. Programa de Pós-Graduação em Saúde Pública. São Paulo, SP, Brazil; II Universidade de São Paulo Instituto de Ciências Biomédicas Departamento de Parasitologia São Paulo SP Brazil Universidade de São Paulo. Instituto de Ciências Biomédicas. Departamento de Parasitologia. São Paulo, SP, Brazil; III University of Florida Florida Medical Entomology Laboratory Vero Beach FL United States of America University of Florida. Florida Medical Entomology Laboratory. Vero Beach, FL, United States of America; IV Universidade de São Paulo Faculdade de Saúde Pública Departamento de Epidemiologia São Paulo SP Brazil Universidade de São Paulo. Faculdade de Saúde Pública. Departamento de Epidemiologia. São Paulo, SP, Brazil; V Universidade de São Paulo Instituto de Medicina Tropical Laboratório de Virologia São Paulo SP Brazil Universidade de São Paulo. Instituto de Medicina Tropical. Laboratório de Virologia. São Paulo, SP, Brazil

**Keywords:** Aedes albopictus, Zika Virus, Locomotor Activity, Infection

## Abstract

**OBJECTIVE:**

To investigate the effect of Zika virus on the locomotor activity of a Brazilian population of *Aedes albopictus* under laboratory conditions.

**METHODS:**

Females of *Aedes albopictus* were infected with Zika virus orally or by intrathoracic injection. The locomotor activity was monitored using a *Drosophila* activity monitor under controlled conditions of 25°C and a 12h light/dark cycle. The infection status was determined using reverse transcription followed by real-time polymerase chain reaction (RT-qPCR). Statistical analyses were conducted using generalized linear mixed models (GLMMs).

**RESULTS:**

The locomotor activities of Zika virus-infected and uninfected *Aedes albopictus* females were diurnal and bimodal, with peaks at lights on and off. The infection did not significantly alter the total activity, diurnal and nocturnal, or the light-on and light-off peaks of infected females compared with uninfected females, regardless of the method of infection (intrathoracic injection or orally).

**CONCLUSION:**

This finding indicates that Zika virus infection does not affect the daily activity pattern of this species under laboratory conditions, which reinforces the importance of this species as a competent and adaptable vector in urban and rural areas, confirming the importance of ongoing surveillance and control strategies.

## INTRODUCTION

Every year millions of individuals worldwide are infected with dengue (DENV), Zika (ZIKV), and chikungunya (CHIKV) arboviruses, particularly in tropical and subtropical regions. The arboviruses are transmitted to humans through the bite of infected female mosquitoes, especially of the *Aedes* genus^1,2^. One of these species is *Aedes* (*Stegomyia*) *albopictus* (Skuse), which is also known as the Asian tiger mosquito, due to its original distribution in Southeast Asia^3^.

This species is a major global invader and has specific characteristics that support its successful expansion to different continents^1,4^. An important characteristic is the drought-resistant eggs, which facilitate its geographical expansion into new regions, including those with a temperate climate, such as North America and Europe, which are easily tolerated by *Ae. albopictus*^5,6^. Additionally, it is an important competent vector of DENV, ZIKV, CHIKV, and YFV in different countries^5^. For example, recent epidemics of CHIKV in France and Italy have identified *Ae. albopictus* as the primary vector^10,11^, and it was also identified as a vector in the Zika outbreak in Gabon^12^.

In Brazil, DENV, ZIKV, and CHIKV are transmitted by the mosquito *Aedes* (*Stegomyia*) *aegypti* (Linnaeus, 1762). *Ae. albopictus*, which is also widely distributed throughout Brazil^13^, is a potential vector of these arboviruses^2^ since it has demonstrated vector competence for the transmission of DENV, ZIKV, and CHIKV in laboratory conditions^14^. Additionally, naturally infected specimens have been found in the wild, especially by vertical transmission^16,17^, which highlights the necessity for enhanced surveillance and control measures for this mosquito in the country^18^.

The daily activities of *Ae. albopictus* occur mainly during the daytime, showing a bimodal pattern, with peaks at the beginning and end of the light phase^19,20^. Biotic and abiotic factors can influence the locomotor activity of mosquito vectors, and one crucial factor is arbovirus infection. For example, infection by DENV-1, DENV-2, and ZIKV alters the locomotor activity of *Ae. aegypti* females^21^.

Vector capacity factors, such as lower anthropophilia and endophilia compared with *Ae. aegypti*, may help in understanding the role of *Ae. albopictus* as a vector in Brazil^16^. However, the effect of arbovirus infection on the locomotor activity of this species, which can directly influence its pathogen transmission and dispersion capabilities, remains largely unexplored. Therefore, this study aims to investigate, for the first time, the effect of ZIKV infection on the locomotor activity of *Ae. albopictus* under controlled laboratory conditions to comprehensively understand the relationship between this virus and invertebrate host.

## METHODS

### Ethical Statement

This study was approved by the ethics committee of the Faculdade de Saúde Pública da Universidade de São Paulo, under Opinion 6.049.131 and CAAE: 68515122.7.0000.5421.

### Mosquito Rearing

*Ae. albopictus* eggs from colonies at *Laboratório de Mosquitos Transmissores de Hematozoários*, Fundação Oswaldo Cruz, state of Rio de Janeiro, Brazil, were hatched in plastic trays (27 × 20 cm) containing 1 L of distilled water and fish food (TetraMin^®^). Each tray contained up to 200 larvae to avoid competition. The pupae were then placed in entomological cages until the adult emergence. Male and female mosquitoes were kept together in the same cages until the beginning of the experiments to ensure that the females were inseminated at the time of infection. Male and female adults were fed a 15% sugar solution, and only females were used in the locomotor activity experiments.

### Intrathoracic Infection

Zika virus was obtained from the Laboratory of Clinical and Molecular Virology (LVCM)-USP and Virology laboratory (IMT-FM), where we confirmed the concentration of viral culture. Inseminated females of *Ae. albopictus*, 8 to 11 days old, were used for infection by intrathoracic injection. After anesthetized at a low temperature, one group of females was inoculated with 0.28 μL of Zika virus culture at a concentration of 10^5^ PFUs, and the other group was inoculated with 0.28 μL of culture medium (control). After intrathoracic injection, each female was individualized in cylindrical glass tubes (1 × 10 cm) with absorbent cotton on both sides, one of which was soaked in a sugar solution to feed the mosquitoes during the days of the experiment. Both ends of the tubes were sealed with Parafilm^®^.

### Oral Infection

Inseminated females of *Ae. albopictus,* 8 to 11 days old, were used for oral infection. They were separated into small cages without sugar one day before the experiment and human blood from a volunteer was used to feed them. The infection by blood-feeding was conducted in the Laboratory of Genetically Modified Mosquitoes at ICB II-USP, in a biosafety level 2 insectary. Then, 500 μL of sodium citrate was added to the human blood, followed by centrifugation at 447 RCF (2,000 rpm) for 5 min at 4°C. The serum was then separated into a new tube, and the red blood cells were maintained at 37°C and the serum at 56°C for 30min. To feed the infected group, 450μL of red blood cells + 450μL of Zika virus culture at a concentration of 10^7^ PFUs + 45μL of serum were used. Only red blood cells and serum were used for the control group. Females were blood-fed using the Hemotek membrane feeding system^24^, which maintains the blood at 37°C for an extended period. The females were anesthetized in CO_2_ after approximately 1h of feeding, and only the engorged females were individualized in the cylindrical glass tubes (1 × 10 cm), as described above.

### Locomotor Activity

The closed tubes containing the inoculated or blood-fed females were placed in a larger model of the *Drosophila* activity monitor (TriKinetics Inc, Waltham, MA, USA). Each activity monitor contains 32 channels equipped with infrared beams that detect and record mosquito movement whenever an individual crosses the beam^21,25^. All monitors were placed in a Precision Scientific Model 818 Incubator under a constant temperature of 25°C and a photoperiod under a 12h light and a 12h dark cycle. For each female, the total locomotor activity during 30-min intervals was continuously recorded for all experimental days after inoculation, using the DAM System Data Collection program (TriKinetics Inc., Waltham, MA, USA). The locomotor activity experiments of intrathoracically infected and orally infected females were performed for seven and eight consecutive days, respectively, and the data were organized using Excel.

All live females were stored in a −80°C ultrafreezer to confirm the infection status of the infected group at the end of the experiment. Live females from the control group were also stored to verify that there was no contamination between the groups.

### Detection of Zika Virus in Mosquitoes

Females from both infection methodologies groups had their heads, thoraxes, and abdomens separated on a cold table. We examined both the head and abdomen, as the injection was administered directly into the thorax, to detect Zika virus in intrathoracically infected females. The head, thorax, and abdomen were examined for females infected by blood feeding.

**Table:** Locomotor activity indices (with standard error) of *Aedes albopictus* females infected with Zika virus, from the intrathoracic infection and oral infection methodologies.

Index	Aedes albopictus - intrathoracic infection			Aedes albopictus - oral infection		
	Status 0 (control) n = 69	Status 1 (infected) n = 48	F test	Status 0 (control) n = 22	Status 1 (infected) n = 31	F-test^a^
I) Total activity	0.829 (0.68)	0.795 (0.57)	F_1, 0.956_ = 0.002; p = 0.974	0.999 (1.20)	0.781 (0.58)	F_1, 3.134_ = 0.683; p = 0.467
II) Diurnal activity	0.878 (0.92)	0.807 (0.68)	F_1, 2.599_ = 0.047; p = 0.844	1.100 (1.08)	0.979 (0.92)	F_1, 26.857_ = 0.226; p = 0.638
III) Diurnal activity without lights on	0.839 (0.90)	0.757 (0.65)	F_1, 2.100_ = 0.027; p = 0.884	1.086 (1.08)	0.956 (0.88)	F_1, 26.471_ = 0.219; p = 0.644
IV) Nocturnal activity	0.853 (0.81)	0.868 (0.86)	F_1, 1.456_ = 0.034; p = 0.876	0.986 (1.51)	0.707 (0.76)	F_1, 2.565_ = 0.750; p = 0.460
V) Nocturnal activity without lights off	0.707 (0.74)	0.729 (0.79)	F_1, 2.971_ = 0.025; p = 0.884	0.860 (1.44)	0.589 (0.74)	F_1, 2.711_ = 0.762; p = 0.453
VI) Lights on activity	3.526 (5.09)	3.286 (4.70)	F_1, 0.637_ = 0.026; p = 0.909	2.054 (2.60)	4.117 (14.57)	F_1, 1.683_ = 0.113; p = 0.774
VII) Lights off activity	15.32 (14.2)	12.30 (9.57)	F_1, 1.751_ = 0.022; p = 0.897	10.66 (8,54)	13.73 (13.53)	F_1, 1.465_ = 0.186; p = 0.721

GLMM: generalized linear mixed models.

^a^Results of the F-test (degrees of freedom, F-value, and p-value) when status was included as a fixed effect in the GLMM.

Viral RNA was extracted using RNAdvance Viral XP (Beckman Coulter, Inc. USA). For RT-qPCR, we used the TaqMan Fast Virus Master Mix enzyme (Thermo Fisher Scientific, Waltham, Massachusetts, USA) with specific primers for *Orthoflavivirus* Flavi all S, Flavi all s2, and Flavi all probe 3^26^. Negative controls were added in each run of RT-qPCR, represented by the females in the control group, and a positive control, represented by RNA from the same Zika virus culture, which was used to infect the females. The runs were made on QuantStudio 5 (Thermo Fisher Scientific, Waltham, Massachusetts, USA).

For both methodologies, we considered that infected females were all those alive by the end of the experiments and had infection confirmed by RT-qPCR (Ct up to 37).

### Statistical Analysis

Statistical analyses were performed using data from the 2^nd^ to the 6^th^ day post-infection, as intrathoracic infection causes the virus to migrate more quickly to the brain and salivary glands of females^21^. We used days 5, 6, and 7 post-infection for statistical analysis for the oral infection method, as the virus passes through mechanical barriers in the female’s digestive tract until it reaches the brain and salivary glands^22^.

Locomotor activity values were transformed into log (N + 1) for statistical analysis to minimize the influence of extreme values—both high and low—and to accommodate zero values, which are commonly observed in mosquito activity. For exploratory analysis and construction of activity figures, William’s mean (Mw)^27^ (a modified geometric mean that accounts for frequent zero values and provides an estimate of the central tendency of activity during each time interval^20^) was applied for each 30-min interval as previously described^21,25^.

To compare the locomotor activity of *Ae. albopictus* females infected with ZIKV and the control group, we initially used generalized linear mixed models (GLMMs) to compare the three experiments for each infection methodology. This confirmed that the experiments were conducted accordingly. Next, we analyzed seven indices related to locomotor activity: (i) total activity; (ii) diurnal activity; (iii) diurnal activity without the lights on period, that is, activity during the photophase except for the first 30 min; (iv) lights on activity, which corresponds to the first 30 min after lights on; (v) nocturnal activity; (vi) nocturnal activity without the lights off period, that is, activity during the scotophase except for the first 30 min; and (vii) lights off activity, which corresponds to the first 30 min after lights off.

We used GLMMs with a Gaussian distribution as we repeated the measurements for each individual mosquito over several days of continuous experiments^28^. Therefore, we used the lmer function from the R package lme4^29^. We created seven models for each index, which included a random effect for mosquito identity to enable repeated measurements.

We included the parameter “status” as a fixed effect, a categorical parameter with two levels: 0 (control group) and 1 (infected group). F-tests were conducted to test whether the inclusion of the fixed effect in the models was significant (based on a type III ANOVA table). The p-values were calculated with the ImerTest package in R using Satterthwaite’s degrees of freedom method^30^for the F-test^31^. All the analyses were conducted in the R (4.2.1) computing environment using the RStudioRStudio 2024.09.1+394 “Cranberry Hibiscus” (R Core Team, 2021).

## RESULTS

A total of 140 and 148 *Ae. albopictus* females were intrathoracically injected in the control and infected groups, respectively. For oral feeding, 57 and 116 females were used for the control and infected groups, respectively. At the end of the experiments, only live females were confirmed to be infected with ZIKV. Of the 62 and 46 females tested, 48 and 31 were infected (77.4% and 69.6%), and the locomotor activity was assessed using the intrathoracic and oral infection methods, respectively. For the control group, locomotor activity was analyzed for 69 and 22 females with intrathoracic and oral infections, respectively.

**Figure 1:**
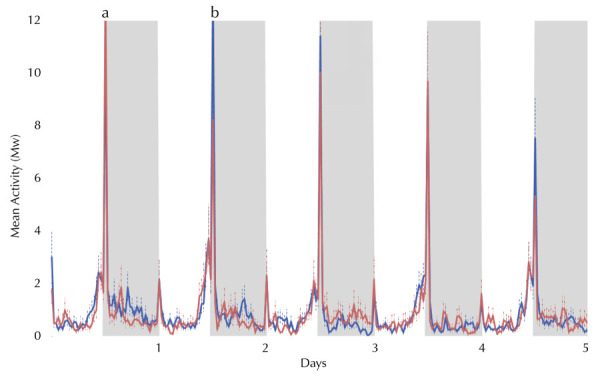
Locomotor activity (William’s mean) of intrathoracically infected *Aedes albopictus* females. DPI: days post infection; LD: lightdark.

Note: control group (n = 69; blue line) and Zika virus-infected group (n = 48; red line) of females exposed to five days under 12h of light (white columns) and 12 h of dark (gray columns) (LD 12:12), at 25°C, from the 2^nd^ DPI to the 6^th^ DPI. The dotted bars represent the standard error of Mw. Some reading time points that exceed the scale are indicated with letters, and their values are as follows: a (infected females) = 13.64 (+1.99); b (control females) = 13.50 (+2.64).

The locomotor activity of intrathoracically infected females between 2 and 6 days post infection (DPI) was diurnal and bimodal, with a lower peak at light-on and a higher peak at light-off (Figure 1). The daytime activity was notably high from the middle of the light phase, immediately before lights off (Figure 1). During the five days of evaluation, the light-off peak of the control females was higher than that of the infected females on the 3^rd^, 4^th^, and 6^th^ DPIs (Figure 1).

**Figure 2:**
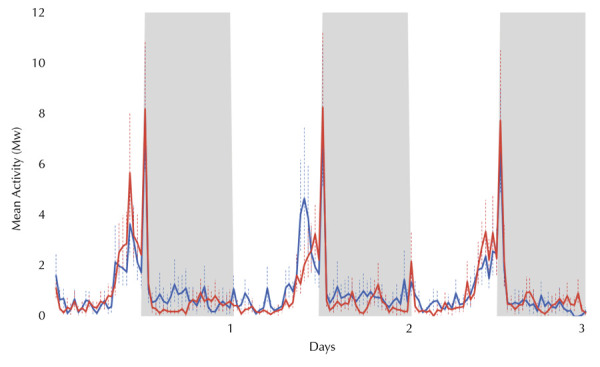
William’s mean locomotor activity (Mw) of orally infected *Aedes albopictus* females. DPI: days post infection; LD: lightdark.

Note: control group (n = 22; blue line) and Zika virus-infected group (n = 31; red line) of females exposed to three days under 12h of light (white columns) and 12h of dark (gray columns) (LD 12:12) at 25°C, from the 5^th^ DPI to the 7^th^ DPI. The dotted bars represent the standard error of Mw.

The uninfected and infected *Ae. albopictus* females showed similar results using the blood-feeding method. The locomotor activity of both groups was bimodal, with a small peak at light-on and a very sharp peak at light-off (Figure 2). Locomotor activity in the light period was also notably high from the middle of the light phase, immediately before lights off (Figure 2). The light-off peak of infected females was higher on all the days evaluated (Figure 2).

No significant statistical differences were observed between the replication of the intrathoracic infection method and the oral infection method. Therefore, the experiments were grouped according to infection methodology for statistical analysis.

For intrathoracic injection, the F-test by GLMMs indicated that Zika virus infection had no significant effect on the locomotor activity of *Ae. albopictus* females, compared with the control group for any of the parameters evaluated: total activity (F_1, 0.956_ = 0.002; p = 0.9737), diurnal activity (F_1, 2.599_ = 0.047; p = 0.844), diurnal activity without lights on (F_1, 2.100_ = 0.027; p = 0.884), nocturnal activity (F_1, 1.456_ = 0.034; p = 0.876), nocturnal activity without lights off (F_1, 2.971_ = 0.025; p = 0.884), lights on activity (F_1, 0.637_ = 0.026; p = 0.909), and lights off activity (F_1,1.751= 0.022_; p = 0.897) (Table).

Similarly, the F-test by GLMMs for oral feeding showed that Zika virus infection had no significant effect on the locomotor activity of the population of this species, compared with the control group for all the parameters tested: total activity (F_1, 3.134_ = 0.683; p = 0.467), diurnal activity (F_1, 26.857_ = 0.226; p = 0.638), diurnal activity without lights on (F_1, 26.471_ =0.219; p = 0.644), nocturnal activity (F_1,2.565_ = 0.750; p = 0.460), nocturnal activity without lights off (F_1, 2.711_ = 0.762; p = 0.453) lights on activity (F_1,1.683_ = 0.113; p = 0.774), and lights off activity (F_1,1.465_ = 0.186; p = 0.721) (Table).

**Figure 3:**
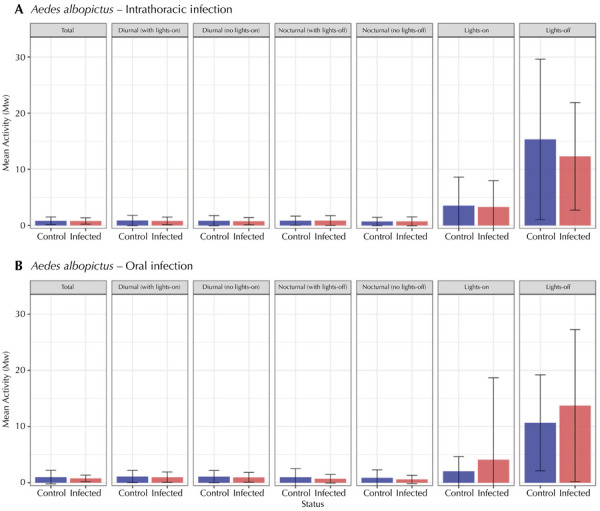
Total activity, daytime activity, daytime activity with lights off, nighttime activity, nighttime activity with lights on and activity with lights on and off of the *Aedes albopictus* females.

Note: the error bars represent the standard errors of the mean. Control group (blue) and infected group (red). A) Intrathoracic infection. B) Oral infection.

The results of the models generated showed that when all the other parameters were controlled, no significant difference was observed between control and infected females (status 0/status 1) in both infection methods for total activity, diurnal activity, diurnal activity without lights on, lights on activity, nocturnal activity, nocturnal activity without lights off, and lights off activity (Figure 3).

## DISCUSSION

To the best of our knowledge, this is the first study to investigate the effect of ZIKV infection on the locomotor activity of a Brazilian population of *Ae. albopictus* under laboratory conditions. Our results showed that ZIKV infection did not significantly affect the locomotor activity of *Ae. albopictus* females. Notably, the same result was observed for intrathoracic injection and blood feeding, which were used for the experimental infection of mosquitoes.

The locomotor activity of ZIKV-infected and uninfected *Ae. albopictus* females were diurnal and bimodal, with peaks when the lights went on and off. This pattern of locomotor activity has also been reported for this species in previous studies under laboratory conditions^20,25,32^.

Abiotic factors, such as temperature, and biotic factors, such as insecticide resistance, physiological state, and viral infections, can affect the locomotor activity of vector mosquitoes, such as *Ae. aegypti* and *Ae. albopictus*^20^. Brazilian populations of *Ae. aegypti* from the southeastern and northeastern regions of the country, with some degree of resistance to pyrethroids, showed significantly higher locomotor activity compared with the population susceptible to this insecticide^33,34^. Laboratory studies have shown that insemination and blood-feeding alone reduce the locomotor activity of *Ae. aegypti* females compared to the control group; however, the same was not observed in *Ae. albopictus* females^20,32^.

Infections with the DENV and ZIKV viruses cause notable changes in the locomotor activity of *Ae. aegypti*^21^. *Ae. aegypti* females of the PAEA strain (Tahiti, French Polynesia) were injected intrathoracically with DENV-2 and a significant increase in locomotor activity was observed, compared with uninfected females^21^. Similarly, *Ae. aegypti* females from a Brazilian colony were orally infected with DENV-1 and showed a significant increase in the overall locomotor activity in the 4, 5, and 6 DPI^23^.

A significant increase in locomotor activity was also observed in an Australian population of *Ae. aegypti* females orally infected with ZIKV compared with uninfected females^36^. However, *Ae. aegypti* females of the PAEA strain who were orally infected with ZIKV showed a significant decrease in diurnal locomotor activity compared with the uninfected females^22^. However, in our study, the lack of effect on the locomotor activity of *Ae. albopictus* females infected with ZIKV may be related to differences between these species, e.g., in relation to vectorial capacity.

The *Ae. albopictus* population used in this study showed susceptibility to ZIKV infection, with the virus present in the head or thorax and abdomen of the females evaluated (48/62 intrathoracic and 31/46 oral infections). The vectorial competence of Brazilian populations of *Ae. albopictus* for the Zika virus has been demonstrated in the laboratory^15^. Although this species has not been identified as a primary vector in Brazil, it transmits CHIKV and ZIKV in several countries of temperate Europe, and Africa^10^.

Several factors contribute to understanding why *Ae. albopictus* is considered a potential vector in Brazil. One aspect regards its ecology as this species is found in areas with relative vegetation cover in Brazil, such as peri-urban, wild environments, and urban parks^16^. In addition, studies have indicated that *Ae. albopictus* has a considerable vectorial capacity and transmission efficiency of ZIKV when exposed to lower temperatures^35,37^. In contrast, this mosquito is less likely to feed on blood at higher temperatures^35,37^. Thus, temperature could be an important factor for *Ae. albopictus* in sustaining arbovirus transmission, as higher temperatures found in most regions of South America could hinder the spread of ZIKV.

Importantly, the lack of a change in the locomotor activity of *Ae. albopictus* females infected with ZIKV can have a positive effect in this arbovirus transmission. *Ae. albopictus* is considered a wilder species with less association with humans compared to *Ae. aegypti*^16^. Therefore, the lack of change in locomotor activity indicates that ZIKV-infected females remain active to seek out a host to feed on blood, e.g., facilitating the pathogen transmission.

Our study demonstrated that regardless of the method of infection used, no changes were observed in the locomotor activity of *Ae. albopictus* females infected with ZIKV compared with uninfected females. The intrathoracic injection may be a less natural and more invasive method, but our results showed no impairment of mosquito structures that could interfere with their locomotor activity or their ability to become infected, consistent with previous reports^21,38^. Although blood feeding is the primary natural route of infection for a female mosquito^22^, the population of *Ae. albopictus* used in our study showed relative resistance to blood feed under laboratory conditions, which limited the number of specimens sampled in our experiments. However, no significant differences were observed between the replicates of the experiments, which confirmed the results.

The methodology used in our study has limitations, e.g., light and dark transitions occur abruptly, which does not occur in nature. However, our results advanced our understanding of the interaction between *Ae. albopictus* and ZIKV. The demonstrated vectorial competence and adaptability to various environmental conditions make *Ae. albopictus* a possible arbovirus transmitter in Brazil^16^.

The ability of *Ae. albopictus* to transit between wild, peri-urban, and urban environments highlights its potential as a link for viruses restricted to natural habitats, such as the yellow fever virus (YFV)^8,18^. Moreover, studies have demonstrated the presence of vertical transmission of DENV and ZIKV in *Ae. albopictus* through naturally infected larvae and males, highlighting the importance of this species in maintaining circulation in wild environments and inter-epidemic periods of these arboviruses^16^. Consequently, control strategies should also prioritize the reduction of the population of this species and its adaptation to new environments, where the mosquito may have greater contact with humans. The interaction between factors, such as the population diversity of *Ae. albopictus* and viral strains of different arboviruses circulating in Brazil, reveals the need for further research to clarify the role of *Ae. albopictus* in arbovirus transmission in the country.

## CONCLUSION

This study is the first to analyze the activity of *Ae. albopictus* infected with ZIKV and aimed to address gaps in the understanding of vector-pathogen interaction. The results indicated that the Brazilian population of *Ae. albopictus* does not have altered locomotor activity when infected with ZIKV compared to uninfected ones, which may help to explain why this species is considered a potential vector in Brazil. Our results highlight the importance of maintaining active entomological surveillance of *Ae. albopictus* in Brazil. Further research is needed to explore the locomotor activity of *Ae. albopictus* females infected with other arboviruses and under different environmental conditions. Comparisons between populations of this mosquito from different regions worldwide and infected with different viral strains are fundamental to understanding the role of *Ae. albopictus* as a vector of arboviruses worldwide, as well as helping to improve and develop more targeted vector control strategies.
